# von Willebrand factor deficiency does not influence angiotensin II-induced abdominal aortic aneurysm formation in mice

**DOI:** 10.1038/s41598-018-35029-8

**Published:** 2018-11-09

**Authors:** Irina Portier, Kimberly Martinod, Linda Desender, Nele Vandeputte, Hans Deckmyn, Karen Vanhoorelbeke, Simon F. De Meyer

**Affiliations:** Laboratory for Thrombosis Research, KU Leuven Campus Kulak Kortrijk, Kortrijk, Belgium

## Abstract

Abdominal aortic aneurysm (AAA) refers to a localized dilation of the abdominal aorta that exceeds the normal diameter by 50%. AAA pathophysiology is characterized by progressive inflammation, vessel wall destabilization and thrombus formation. Our aim was to investigate the potential involvement of von Willebrand factor (VWF), a thrombo-inflammatory plasma protein, in AAA pathophysiology using a dissection-based and angiotensin II infusion-induced AAA mouse model. AAA formation was induced in both wild-type and VWF-deficient mice by subcutaneous implantation of an osmotic pump, continuously releasing 1000 ng/kg/min angiotensin II. Survival was monitored, but no significant difference was observed between both groups. After 28 days, the suprarenal aortic segment of the surviving mice was harvested. Both AAA incidence and severity were similar in wild-type and VWF-deficient mice, indicating that AAA formation was not significantly influenced by the absence of VWF. Although VWF plasma levels increased after the infusion period, these increases were not correlated with AAA progression. Also detailed histological analyses of important AAA hallmarks, including elastic degradation, intramural thrombus formation and leukocyte infiltration, did not reveal differences between both groups. These data suggest that, at least in the angiotensin II infusion-induced AAA mouse model, the role of VWF in AAA pathophysiology is limited.

## Introduction

Abdominal aortic aneurysm (AAA) is a pathological condition characterized by permanent dilation of the abdominal aorta, affecting 4–7% of men over the age of 55 years^1^. The lethal nature of this pathology is directly linked with the inherent risk of rupture, which has a mortality rate of 50–80%^2^. To avoid rupture, clinically relevant aneurysms that exceed the normal aortic diameter by >50% need regular monitoring until elective surgical repair is indicated. Pharmaceutical treatments are currently lacking, partly due to our limited understanding of the mechanisms underlying AAA pathophysiology. Progressive inflammation, proteolytic degradation, progressive vessel wall destabilization and activation of hemostasis with formation of an intraluminal thrombus have all been identified as hallmarks of AAA progression^3^.

von Willebrand factor (VWF) is a multimeric glycoprotein, produced by endothelial cells and megakaryocytes. VWF is implicated in the regulation of several processes, including thrombosis, hemostasis, vascular stability and inflammation, which together could influence AAA development^4^. Elevated VWF levels have been reported in patients with aortic aneurysms^5^ and in patients with ruptured AAAs^6^. Additionally, VWF activity was recently found to correlate with intraluminal thrombus volume in AAA and hence has been suggested as a possible biomarker for AAA growth^7^. Besides the well characterized thrombo-inflammatory function^8,9^, increasing evidence suggests a role of VWF in several vascular processes. von Willebrand disease, an inherited bleeding disorder caused by quantitative or qualitative defects in VWF, has also been associated with different vascular abnormalities, including arterial dissection^10^, arterial pseudoaneurysms^11–13^, gastro-intestinal angiodysplasia^14^, arteriovenous malformations^15^, and telangiectasia^16^. Furthermore, VWF-deficient mice were shown to display enhanced angiogenesis^[17,18^ and increased vascular density^17^, while vascular smooth muscle cell coverage, characteristic for arterial maturation, during vascular development was delayed^19^.

Whether the different functions of VWF are involved in AAA pathophysiology is currently not known and experimental *in vivo* studies addressing this question are lacking. Therefore, the aim of this study was to investigate the potential involvement of VWF in AAA pathophysiology using an AAA mouse model. A dissection-based AAA model induced by angiotensin II was chosen, as this model is characterized by leukocyte infiltration, elastin degradation and thrombus formation^20^.

## Results

### Incidence and severity of AngII-induced AAA

**Figure 1:**
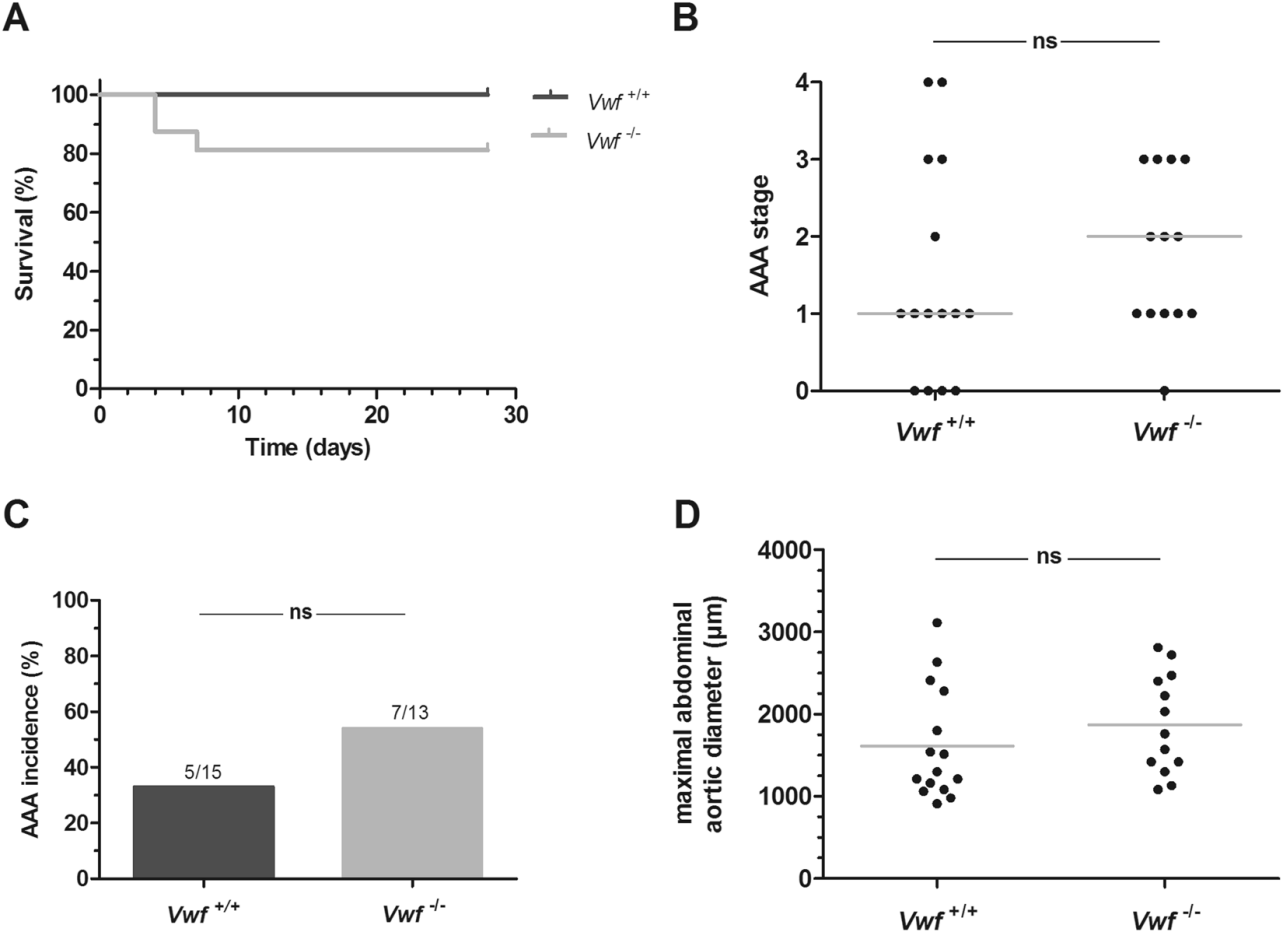
Incidence and severity of AngII-induced AAA formation. *Vwf*^−/−^ and *Vwf*^+/+^ mice were continuously infused with AngII (1000 ng/kg/min) for 28 days via subcutaneously implanted osmotic mini-pumps. (**A**) Kaplan-Meier curves represent the survival in AngII-infused *Vwf*^−/−^ (grey; n = 16) and *Vwf*^+/+^ (black; n = 15) mice. p = 0,083 using the Log-Rank (Mantel-Cox) test. After 28 days, the suprarenal regions were harvested to determine (**B**) the aneurysm stage of the abdominal aorta (p = 0.439; Chi-square test for trend) (**C**) AAA incidence (p = 0.445; Fisher’s exact test) and (**D**) the maximal abdominal aortic diameter (p = 0.301; unpaired t-test). AAA was classified as follows: Stage 0: no dilation; Stage 1: hypertrophy of the adventitia; Stage 2: dilation of the abdominal aorta with or without the presence of a thrombus; Stage 3: pronounced bulbous form of Stage 2; Stage 4: multiple, complex form of Stage 3. Grey lines in graphs of the maximal abdominal aortic diameter and aneurysm staging represent respectively, mean and median values. ns, not statistically significant

To investigate the potential contribution of VWF in AAA pathogenesis, both VWF-deficient (*Vwf*^−/−^) and *Vwf*^+/+^ mice were subjected to an angiotensin II-induced model of aneurysm formation. Mice were continuously infused with angiotensin II (AngII, 1000 ng/kg/min) for 28 days via subcutaneous osmotic mini-pumps. In the *Vwf*^−/−^ group, 3 out of 16 mice (19%) did not reach the end of the 28 day-infusion period. This was presumably due to an aortic rupture (2 mice at day 4 and 1 mouse at day 7) since post-mortem analysis revealed the presence of large amounts of blood in the thoracic and/or abdominal cavity (Fig. 1A). In comparison, all *Vwf*^+/+^ mice (n = 15) survived the 28 day-infusion period without complications. This difference was, however, not statistically significant (p = 0.083, Log-Rank Mantel-Cox test; Fig. 1A).

After the 28 day-infusion period, mice were euthanized and dissected to study AAA formation. As commonly observed in this model, macroscopic analysis of the isolated aortas revealed dilations in the suprarenal part of the abdominal aortas in several *Vwf*^+/+^ and *Vwf*^−/−^ mice. Thoracic abdominal aneurysms were only observed in two *Vwf*^+/+^ and one *Vwf*^−/−^ mice and were, given this low incidence, not further considered. The suprarenal aortic parts were harvested from all mice for histological examination and AAA scoring via H&E staining. To test whether VWF deficiency would affect AngII-induced AAA progression, the severity of AAA formation was scored using a classification system ranging from 0 (no dilation) to 4 (multiple, complex aneurysm with bulbous thrombus) (Supplemental Fig. S1). All stages of AAA severity were observed in *Vwf*^+/+^ mice, whereas the most severe stage 4 was absent in *Vwf*^−/−^ mice (Fig. 1B). Nevertheless, overall severity was not significantly different between the two groups, with a median AAA stage of 2 in the *Vwf*^−/−^ group and 1 in the *Vwf*^+/+^ group. Additionally, the overall AAA incidence (defined as stage 2 or higher) was also not statistically different between *Vwf*^−/−^ mice (7/13; 54%) and *Vwf*^+/+^ animals 5/15 (33%) (Fig. 1C). Accordingly, also the maximal abdominal aortic diameter in *Vwf*^+/+^ animals (1613 ± 683 µm) was comparable to the maximal diameter in *Vwf*^−/−^ animals (1872 ± 605 µm, p = 0.301; Fig. 1D).

Measurement of VWF plasma levels in *Vwf*^+/+^ mice 28 days after initiation of AngII infusion revealed a significant increase compared to baseline levels (169.2 ± 45.1%, p = 0.0001; Supplemental Fig. S2A). However, levels of VWF did not correlate with AAA staging (p = 0.844; Supplemental Fig. S2B). In addition, the activity of the VWF cleaving enzyme ADAMTS13 was measured in the *Vwf*^+/+^ mice, but did not change significantly compared to baseline samples (113.5 ± 18.2%, p = 0.053; Supplemental Fig. S2C) and were also not correlated with AAA severity (p = 0.962; Supplemental Fig. S2D).

**Figure 2:**
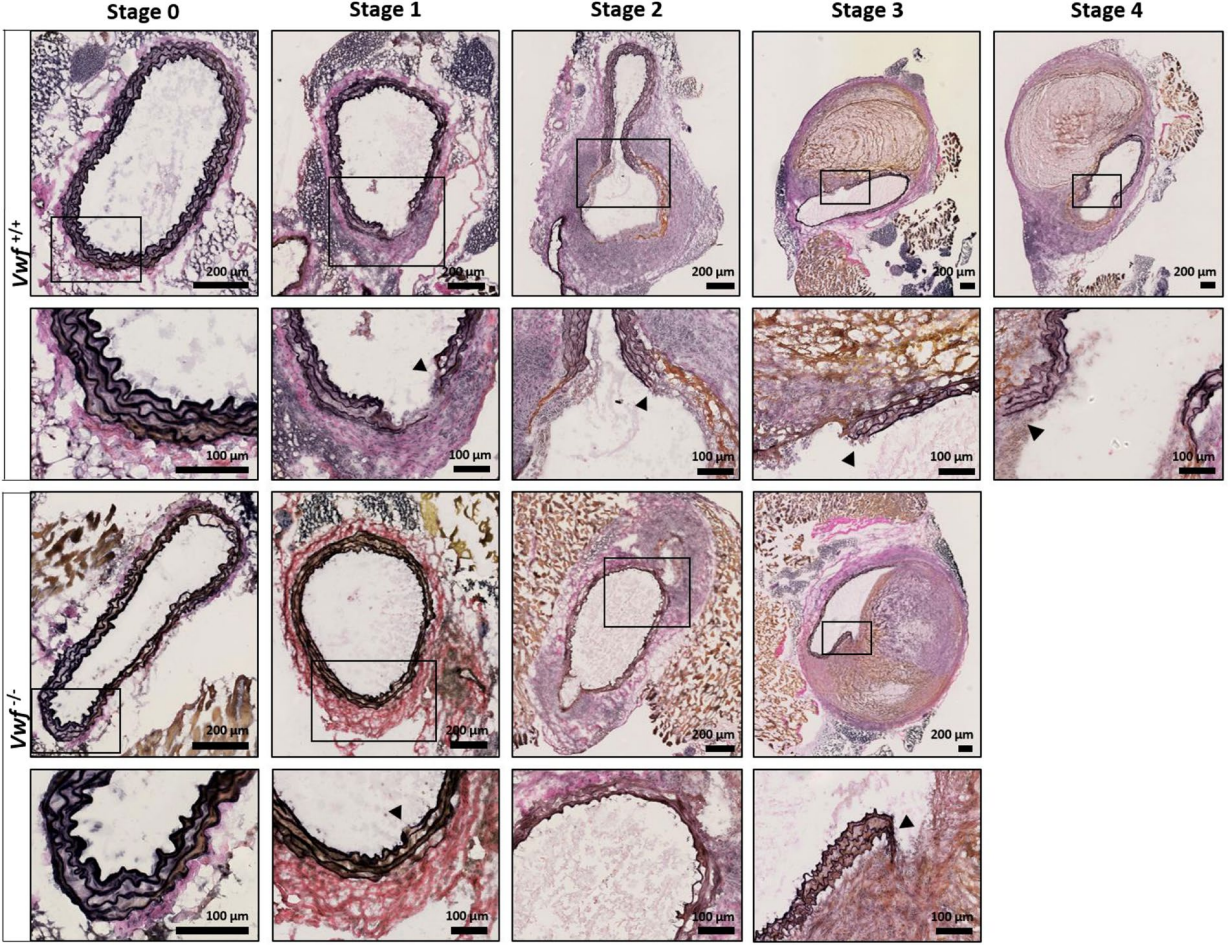
Modified Verhoeff-Van Gieson staining of the elastic laminae in the tunica media. Cryosections of the suprarenal aortic segment of *Vwf*^+/+^ and *Vwf*^−/−^ mice, continuously infused with AngII for 28 days, were stained for elastin using a Modified Verhoeff-Van Gieson staining. Representative sections of every aneurysm stage per group are depicted and the framed areas are shown in greater detail below the original picture. The elastic laminae are visualized by the dark purple lines in the media of the aortas and elastin breaks are indicated by an arrowhead. Collagen appears red/purple on the modified Verhoeff-Van Gieson staining. Images were acquired with a Hamamatsu NanoZoomer-SQ digital slide scanner.

To further investigate potential effects of VWF on AAA progression, modified Verhoeff-Van Gieson elastic staining (Fig. 2) was performed to more specifically visualize the elastin layers in the media. While intact elastin layers could be observed in both groups in mice without AAA development (stage 0), dysregulated elastin layers, including elastin breaks, were visible as early as the precursor stage of AAA (stage 1 and higher). No difference, however, was observed between *Vwf*^−/−^ and *Vwf*^+/+^ animals. In conclusion, these experiments indicate that the absence of VWF does not significantly influence the formation or development of AngII-induced AAA.

### Intramural thrombus formation

**Figure 3:**
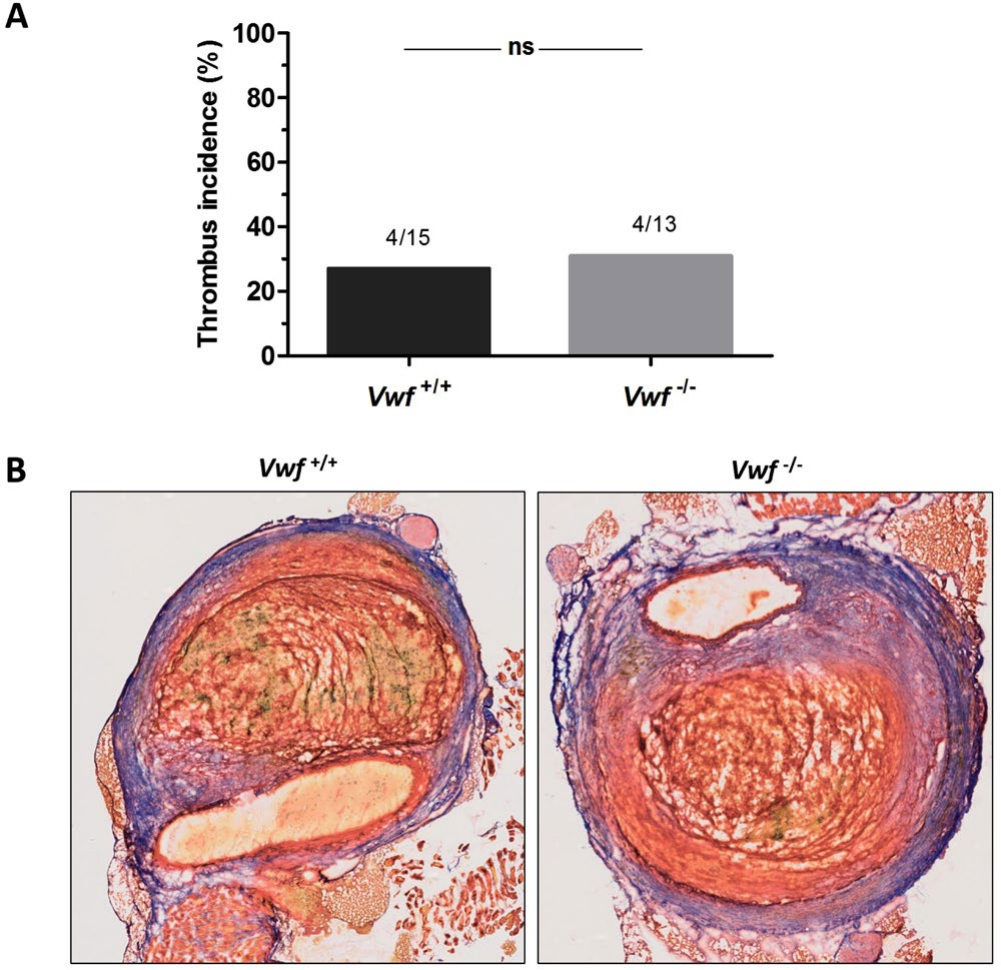
Intramural thrombus formation after AngII-induced AAA formation. *Vwf*^−/−^ and *Vwf*^+/+^ mice were continuously infused with AngII (1000 ng/kg/min) for 28 days via subcutaneously implanted mini-pumps. (**A**) The incidence of thrombus formation was determined after 28 days (p = 1.000; Fisher’s exact test) (**B**) Cryosections of suprarenal aneurysms containing an intramural thrombus (Stage 3–4) were stained for fibrin (red), red blood cells (yellow) and collagen (blue) using Martius Scarlet Blue staining. One representative section per group is depicted and demonstrates that both *Vwf*^+/+^ and *Vwf*^−/−^ mice could form an intramural thrombus with similar compositions. Pictures were taken using a Hamamatsu NanoZoomer-SQ digital slide scanner.

**Figure 4:**
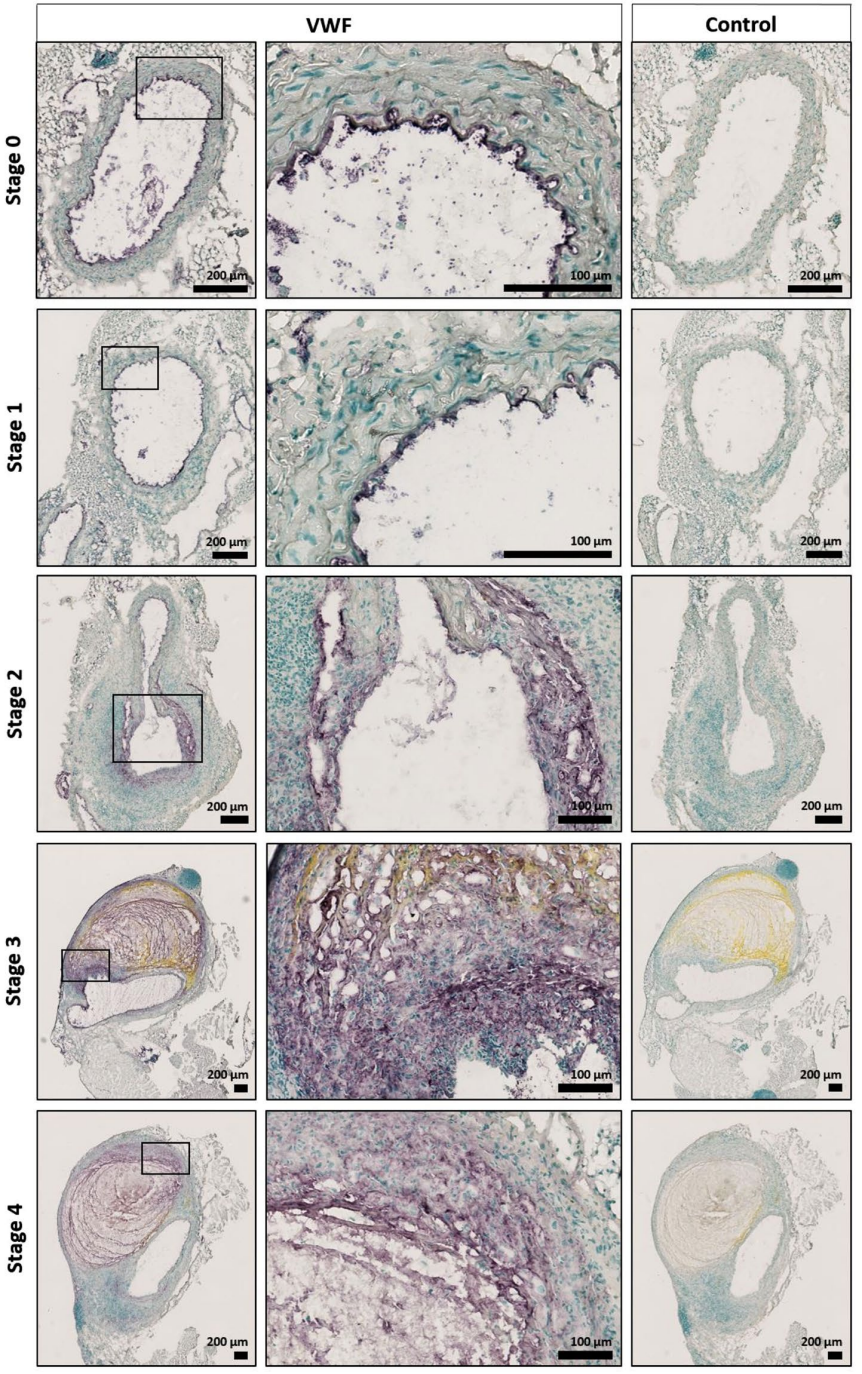
VWF content in the suprarenal aortic tissue of *Vwf*^+/+^ mice after AngII-induced AAA formation. Histological examination of the VWF content in cryosections of the suprarenal aortic segments of *Vwf*^+/+^ mice, which received AngII for 28 days. VWF was stained using rabbit anti-VWF antibodies (purple color). Cryosections stained without primary antibody were used as negative controls. Nuclei were visualized using a methyl green counter stain (green color). Representative sections of every aneurysm stage per group are depicted and the framed areas are shown in greater detail below the original picture. Pictures were taken using a Hamamatsu NanoZoomer-SQ digital slide scanner.

VWF activity was previously found to be positively correlated with thrombus volume in AAA^7^. To more specifically assess AAA thrombus formation, we used Martius Scarlet Blue (MSB) and VWF staining to investigate thrombus formation (Fig. 3). With MSB, fibrin stains red, red blood cells stain yellow, and collagen stains blue. In accordance with the H&E staining, the aneurysms in 4/15 *Vwf*^+/+^ mice (stages 3–4) had an intramural thrombus containing fibrin and red blood cells. As expected, immunostaining for VWF revealed the presence of VWF in thrombi of *Vwf*^+/+^ animals (Fig. 4). Interestingly, also 4/13 *Vwf*^−/−^ mice (stage 3) developed an intramural thrombus despite the lack of VWF. Measurement of both the fibrin and RBC content in these thrombi via color-based threshold analysis showed no significant differences between *Vwf*^+/+^ (Fibrin, 52.75 ± 16.80%; RBC, 26.33 ± 17.67%) and *Vwf*^−/−^ mice (Fibrin, 63.35 ± 12.21%, p = 0.343; RBC, 4.55 ± 3.92%, p = 0.114; Supplemental Fig. 3A). These results indicate that VWF is not crucial for AAA thrombus formation and that absence of VWF does not change overall thrombus presence and structure.

### Inflammatory infiltration

**Figure 5:**
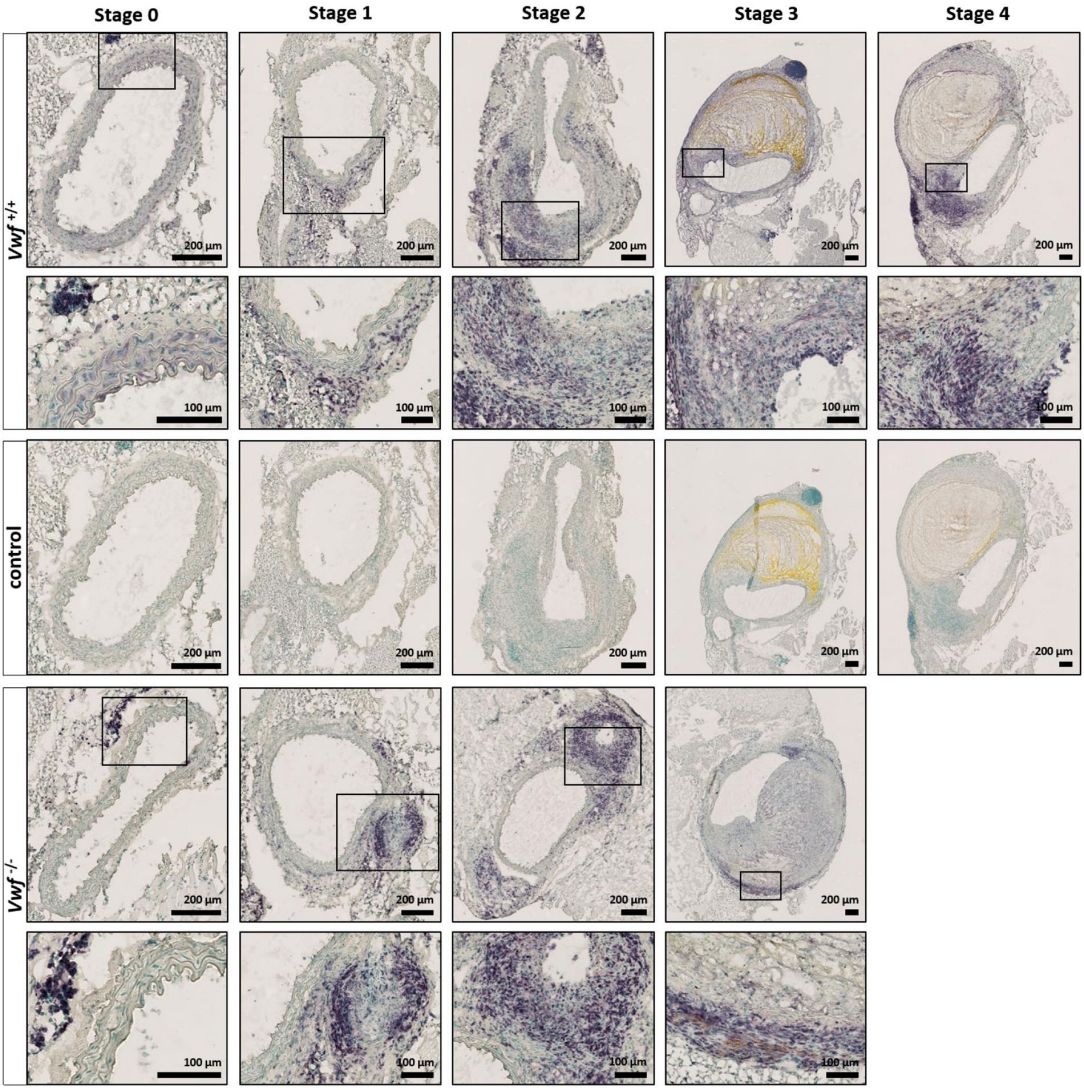
Histological analysis of leukocyte infiltration after AngII-induced AAA formation. Cryosections of the suprarenal aortic segments of *Vwf*^+/+^ and *Vwf*^−/−^ mice, continuously infused with AngII for 28 days, were stained for leukocytes using rat anti-CD45 antibodies (purple color). Nuclei were visualized using a methyl green counter stain (green color). Representative sections of each aneurysm stage per group are depicted and the framed areas are shown in greater detail below the original picture. Cryosections stained without primary antibody were used as negative controls and are shown for the *Vwf*^+/+^ mice. Infiltration of leukocytes was associated with dilating aortic tissue and predominantly present in the adventitia. Pictures were taken using a Hamamatsu NanoZoomer-SQ digital slide scanner.

Increasing evidence suggests that VWF is an important mediator of inflammation. Since AngII-induced AAA formation is associated with infiltration of leukocytes, we performed an immunohistochemical staining of the suprarenal part of the abdominal aortas using the leukocyte marker CD45 (Fig. 5). Results indicate that in both *Vwf*^−/−^ and *Vwf*^+/+^ mice, dilating aortic tissue was associated with infiltration of leukocytes, predominantly into the adventitia (stage 1 and 2). In bulbous aneurysms (stage 3 or higher) leukocytes tended to be marginated to the outer boundaries around the thrombus, although some diffuse immunostaining was present in the remodeled area. To quantify leukocyte infiltration into the suprarenal aortic tissue, the percentage of CD45 positive staining was determined by colour-based threshold analysis. The mean percentage of CD45 positive staining in the *Vwf*^−/−^ group (8.28 ± 4.10%; n = 13) was not significantly different compared to the value in *Vwf*^+/+^ mice (8.99 ± 8.28%; p = 0.782; n = 15; Supplemental Fig. 3B). In conclusion, these experiments indicate that infiltration of inflammatory cells into the aortic tissue occurred to a similar extent in both *Vwf*^−/−^ and *Vwf*^+/+^ mice.

## Discussion

In this study, we investigated the potential effect of VWF on AAA pathophysiology using an AngII-induced mouse model of AAA development. AAA formation was observed in both *Vwf*^+/+^ and *Vwf*^−/−^ mice and no differences were observed in survival, AAA incidence, severity and several histological parameters.

Diagnosis of AAA is difficult, since aneurysms often remain asymptomatic until rupture. Implementation of large ultrasound screening programs to detect and monitor aneurysms has partially reduced rupture-related mortality^21^. However, considering the absence of accurate non-imaging diagnostic methods, prognostic biomarkers for AAA would be of great value. Plasma proteins associated with thrombosis, haemostasis and fibrinolysis have been commonly investigated as potential biomarkers for AAA. Increased levels of VWF have been reported in patients with aortic aneurysm^5^ and in patients with ruptured AAAs^6^. Similarly, ADAMTS13, the VWF activity-reducing enzyme, has been shown to be lower in AAA patients^22^. In a recent report, Ghulam *et al*. showed a positive correlation between VWF activity in plasma and AAA thrombus volume^7^. Since thrombus growth is assumed to influence AAA growth and rupture risk, a link between VWF, thrombus growth and AAA rupture could become relevant. However, other studies did not find a difference in VWF levels between healthy volunteers and patients with AAA and also found no association between VWF and AAA size^22–26^. Despite an increase in VWF plasma levels after AngII infusion, our present study in mice did not find a significant direct effect of VWF on AngII-induced aneurysm formation and progression. Although these animal experiments suggest no causal role between increased VWF and AAA development, more human prospective studies are needed to establish whether increased VWF levels in patients is cause or consequence.

The AngII-induced AAA model is a dissection-based mouse model widely used to study AAA pathogenesis. The pathophysiology of this model is characterized by an initial dissection of the intima and media in the suprarenal aortic segment within the first days after starting continuous AngII infusion. Dissection results in an intramural hematoma with subsequent dilation of the aortic tissue.^27^ Additionally, elastin degradation and leukocyte infiltration have been described as important characteristics in this model^20^. In contrast to other cardiovascular diseases, e.g. ischemic stroke^28–30^, deep vein thrombosis^31^ and myocardial infarction^32,33]^, the thrombo-inflammatory function of VWF does not seem to play a role in AngII-induced AAA. Notably, in a recent report we demonstrated that *Vwf*^-/-^ mice subjected to the AngII-infusion model had reduced cardiac fibrosis, both perivascularly and in the myocardial interstitium^34^.

Although mouse models provide valuable insights into AAA pathophysiology, the direct translational value for the clinic has limitations. The choice of the AngII model in this study was based on the importance of thrombus formation and inflammation in this model. However, other AAA experimental models might also be valuable since different animal models reflect different aspects of human AAA pathogenesis^35^ In addition, normocholesterolemic mice were used in this study. Although the AngII-infusion model is known to induce AAA formation in normocholesterolemic mice^36^, the incidence of AAAs is higher when hypercholesterolemic *ApoE*^−/−^ mice are used. Pre-existing hyperlipidemia and atherosclerosis in *ApoE*^−/−^ mice facilitate AngII-induced AAA formation^36^. Given the involvement of VWF in atherosclerosis^37^, AngII infusion in *Vwf*^−/−^*ApoE*^−/−^ double KO mice could be of interest in future studies, potentially increasing the sensitivity for VWF-mediated mechanisms in AAA.

In conclusion, we have demonstrated that VWF-deficiency does not influence AAA pathogenesis, at least in the AngII-induced mouse model. Based on survival, AAA incidence, elastin degradation, intramural thrombus formation and inflammation, we conclude that AngII-induced AAA formation occurred in a similar fashion regardless of the presence of VWF.

## Materials and Methods

### Mice

Male VWF-deficient (*Vwf*^−/−^)^38^ and *Vwf*^+/+^ mice on a C57BL/6J background (in-house bred) were used in this study. All animal experiments were approved by the Institutional Animal Care and Use Committee of the KU Leuven (Belgium) and performed in accordance with the local ethical law, the regulations of the local ethical committee and the guidelines for the care and use of laboratory animals.

### Angiotensin II induced AAA model

Male mice (16–17 weeks old) received 1000 ng/kg/min angiotensin II (AngII; Sigma-Aldrich, Saint Louis, MO) for 28 days via subcutaneously implanted osmotic mini-pumps (ALZET Model 2004, Charles River, L’Abresle, France) as previously described^39^. Briefly, mice were anesthetized using 2.5% isoflurane (Piramal Healthcare, Morpeth, UK) in 100% O_2_ and placed on a heating pad. Osmotic mini-pumps containing AngII dissolved in sterile saline were implanted in the subcutaneous space along the dorsal midline. Post-operative analgesia (buprenorphine, 0.1 mg/kg) was administered via subcutaneous injection prior to surgery and subsequently every 12 h for 72 h. All mice were monitored daily throughout the experimental period to observe fatalities or to euthanize mice that reached the humane endpoint.

### Tissue collection

Mice were sacrificed 28 days after implantation of the osmotic mini-pumps and perfused with phosphate buffered saline (PBS) through the left ventricle of the heart. Aortas were harvested and the suprarenal parts were snap frozen in Optimal Cutting Temperature compound and stored at −20 °C until processing. Mice that died before the 28 day end point underwent post-mortem analysis to determine if cause of death was aneurysm-related.

### Histological procedures

Nine micrometer cross-sections of aortic tissue were obtained using a Leica CM 1950 cryostat (Leica Biosystems, Nussloch, Germany) and stored at −20 °C prior to histological analysis. Cryosections were stained with hematoxylin and eosin (H&E; Sigma-Aldrich) or Modified Verhoeff Van Gieson Elastic Stain Kit (Sigma-Aldrich) according to the manufacturer’s instructions. Martius Scarlet Blue (MSB) staining was performed as previously described^40^. Additionally, immunohistochemical staining was performed for VWF and leukocytes. After fixation with 4% paraformaldehyde (Merck, Darmstadt, Germany) and washing with Tris-buffered saline (TBS) with 0.1% Polysorbate 20 (Sigma-Aldrich), cryosections were incubated with blocking solution containing 1% bovine serum albumin (Albumin Fraktion V; Carl Roth, Karlsruhe, Germany), 10% normal serum (either normal swine serum [Jackson ImmunoResearch Laboratories, West Grove, PA] or normal rabbit serum [Dako, Glostrup, Denmark]) and 0.1% Polysorbate 20 for 1 h at room temperature (RT). Sections were incubated overnight at 4 °C with the primary antibody (a polyclonal rabbit anti-human (h)VWF antibody [1:1500; Dako] or a purified rat anti-mouse CD45 antibody [1:25; BD Pharmingen, Franklin Lakes, NJ]). After washing, endogenous peroxidase was blocked using a TBS solution containing 0.3% H_2_O_2_. Following consecutive washing steps, the Avidin/Biotin Blocking Kit (Vector Laboratories, Burlingame, CA) was applied according to the manufacturer’s instructions. Next, sections were incubated with biotin-labeled secondary antibodies (swine anti-rabbit-Ig antibody [1:500; Dako] or rabbit anti-rat-IgG antibody [1:100; Vector Laboratories]) for 1 h at RT. After washing, both the VECTASTAIN Elite ABC-HRP Kit (Vector Laboratories) and the VECTOR VIP Peroxidase Substrate Kit (Vector Laboratories) were used for detection according to the manufacturer’s instructions. Counterstaining was performed using VECTOR Methyl Green (Vector Laboratories) according to the manufacturer’s instructions. After dehydration, slides were mounted using Sub-X Mounting Medium (Leica Biosystems, Wetzlar, Germany) and visualized using a NanoZoomer-SQ digital slide scanner (Hamamatsu, Shizuoka, Japan). Color-based threshold analysis was performed using ImageJ software (National Institutes of Health, Bethesda, MD) to quantify the CD45, fibrin and RBC content. All three parameters were defined as the percentage of positive staining compared to the total area of the aortic tissue.

### Classification of AAA stages

Classification of the different forms of aneurysms was done according to a scale that is based on the gross appearance of the abdominal aorta, with slight modifications^41^. Stage 0: no dilation. Stage 1: hypertrophy of the adventitia. Stage 2: small aneurysm with clearly remodeled tissue in the suprarenal region and the maximal abdominal aortic diameter exceeds 1.5 times the normal aortic diameter. In this type, a thrombus can be either present or absent. Stage 3: pronounced bulbous aneurysm that contains a thrombus. Stage 4: complex form of stage 3 with multiple aneurysms containing a thrombus. These aneurysms can overlap in the suprarenal area of the aorta. The maximal diameter of the abdominal aortas was determined on H&E stained cryosections by measurement of the maximal diameter using NDP.view2 software (Hamamatsu). H&E stained sections were chosen approximately 300 µm apart and comprised the full length of the suprarenal aortic segment of AngII-infused mice. The section with the largest maximal abdominal aortic diameter per mouse was chosen as representative cryosection and adjacent slides were used to perform the other stainings.

### Blood collection

Two weeks before and 4 weeks after implantation of the osmotic pumps, blood samples were collected. After anesthesia of mice with 5% isoflurane (Piramal Healthcare) in 100% O_2_, blood withdrawal was performed by retro-orbital puncture and collected into 3.8% trisodium citrate (1 volume to 6 volumes of blood). Platelet poor plasma was prepared by centrifugation at 4,300 g for 6 minutes and immediately stored at −80 °C for further analysis.

### VWF antigen

VWF antigen (VWF:Ag) levels in plasma were determined using an in-house developed enzyme-linked immunosorbent assay as described^42^. Briefly, a microtiter plate was coated with polyclonal anti-human VWF antibody (Dako, Glostrup, Denmark), known to cross-react with mVWF. Captured mVWF was detected using a mixture of in-house generated biotinylated anti-mVWF monoclonal antibodies and horseradish peroxidase-labeled streptavidin. Visualization was obtained with H_2_O_2_ and ortho-phenylenediamine. Pooled plasma from 38 *Vwf*^+/+^ mice was used as a normal murine plasma pool (NMP) reference and results were calculated and expressed as percentage of VWF level compared to the mean of the baseline samples (100%).

### ADAMTS13 activity test

ADAMTS13 ((A Disintegrin-like And Metalloprotease with ThromboSpondin type 1 motif 13) activity in murine plasma samples was measured using the fluorescent resonance energy transfer (FRET) assay with the fluorescent FRETS-VWF73 substrate (Peptides International; Louisville, KY) as previously described^43^. Briefly, 4 µl of plasma was incubated with 2 µM of the FRETS-VWF73 substrate in a HEPES-buffered saline (HBS) solution containing 0.1% bovine serum albumin (Sigma-Aldrich). Proteolysis of the FRETS-VWF73 by plasma ADAMTS13 generates a fluorescent signal that was measured during 60 cycles of 300 s using a FLUOstar OPTIMA reader (BMG Labtech GmbH, Offenburg, Germany) with excitation at 355 nm and emission at 460 nm. Results were expressed as percentage of ADAMTS13 activity compared to the mean of the baseline samples (100%).

### Statistical analysis

All statistical analyses were performed using Prism 5.04 (GraphPad Software; La Jolla, CA). Log-Rank (Mantel-Cox) test was used to compare survival between groups. AAA incidence was analyzed using a Fisher’s exact test on a contingency table containing the absolute values. A Chi-square test for trend was applied to compare AAA stage incidence. Normal distribution of the datasets was tested using D’Agostino & Pearson omnibus normality test. Normally distributed datasets were compared using the unpaired Student’s t-test. Except for differences in ADAMTS13 activity were tested using a paired t-test. Non-normally distributed datasets were compared using the Mann-Whitney test. Except for VWF:Ag levels, which were analyzed using a Wilcoxon matched pairs signed rank test. Finally, Spearman correlation was applied to determine whether VWF:Ag or ADAMTS13 activity were correlated with AAA staging. A level of significance of less than 0.05 was considered in all statistical analyses.

## Electronic supplementary material

Supplemental materials

## Data Availability

The data in this study are available upon request from the corresponding author.

## References

[1] Moll FL (2011). Management of abdominal aortic aneurysms clinical practice guidelines of the European society for vascular surgery. Eur J Vasc Endovasc Surg.

[2] Kuivaniemi H, Ryer EJ, Elmore JR, Tromp G (2015). Understanding the pathogenesis of abdominal aortic aneurysms. Expert Rev Cardiovasc Ther.

[3] Kent KC (2014). Clinical practice. Abdominal aortic aneurysms. N. Engl. J. Med..

[4] Rauch A (2013). On the versatility of von Willebrand factor. Mediterr J Hematol Infect Dis.

[5] Ihara A (2006). Relationship between hemostatic markers and platelet indices in patients with aortic aneurysm. Pathophysiol. Haemost. Thromb..

[6] Skagius E, Siegbahn A, Bergqvist D, Henriksson A (2008). Activated coagulation in patients with shock due to ruptured abdominal aortic aneurysm. Eur J Vasc Endovasc Surg.

[7] Ghulam QM (2016). von Willebrand Factor and Prekallikrein in Plasma Are Associated With Thrombus Volume in Abdominal Aortic Aneurysms. Vasc Endovascular Surg.

[8] Denis CVV, Lenting PJ (2012). von Willebrand factor: at the crossroads of bleeding and thrombosis. Int. J. Hematol..

[9] Kawecki C, Lenting PJ, Denis CV (2017). von Willebrand factor and inflammation. J. Thromb. Haemost..

[10] Rehman AU, Almanfi A, Nadella S, Sohail U (2014). Isolated spontaneous celiac artery dissection in a 47-year-old man with von Willebrand disease. Tex Heart Inst J.

[11] Garaci FG (2003). Hepatic artery pseudoaneurysm in von Willebrand’s disease. Eur Radiol.

[12] Ricciardo BJ, Mwipatayi BP, Abbas M, Sieunarine K, Eikelboom JW (2005). Von Willebrand disease associated with superficial temporal artery pseudoaneurysm. Eur J Vasc Endovasc Surg.

[13] Shimizu M (2011). Secondary postpartum hemorrhage due to uterine artery pseudoaneurysm rupture in von Willebrand disease. J. Obstet. Gynaecol. Res..

[14] Makris M (2015). The natural history of occult or angiodysplastic gastrointestinal bleeding in von Willebrand disease. Haemophilia.

[15] Osenbach RK (1989). Management of intraventricular haemorrhage secondary to ruptured arteriovenous malformation in a child with von Willebrand’s disease. J. Neurol. Neurosurg. Psychiatry.

[16] Conlon CL, Weinger RS, Cimo PL, Moake JL, Olson JD (1978). Telangiectasia and von Willebrand’s disease in two families. Ann. Intern. Med..

[17] Starke RD (2011). Endothelial von Willebrand factor regulates angiogenesis. Blood.

[18] Xu H (2017). ADAMTS13 controls vascular remodeling by modifying VWF reactivity during stroke recovery. Blood.

[19] Scheppke L (2012). Notch promotes vascular maturation by inducing integrin-mediated smooth muscle cell adhesion to the endothelial basement membrane. Blood.

[20] Saraff K, Babamusta F, Cassis LA, Daugherty A (2003). Aortic dissection precedes formation of aneurysms and atherosclerosis in angiotensin II-infused, apolipoprotein E-deficient mice. Arterioscler. Thromb. Vasc. Biol..

[21] Nordon IM, Hinchliffe RJ, Loftus IM, Thompson MM (2011). Pathophysiology and epidemiology of abdominal aortic aneurysms. Nat Rev Cardiol.

[22] Innami Y (2014). Increased prothrombotic property as a risk factor of acute kidney injury after surgical repair of abdominal aortic aneurysm: a prospective observational study. J Intensive Care.

[23] Lee AJ, Fowkes FG, Lowe GD, Rumley A (1996). Haemostatic factors, atherosclerosis and risk of abdominal aortic aneurysm. Blood Coagul. Fibrinolysis.

[24] Blann AD, Devine C, Amiral J, McCollum CN (1998). Soluble adhesion molecules, endothelial markers and atherosclerosis risk factors in abdominal aortic aneurysm: a comparison with claudicants and healthy controls. Blood Coagul. Fibrinolysis.

[25] Fowkes FG (2006). Reduced lung function in patients with abdominal aortic aneurysm is associated with activation of inflammation and hemostasis, not smoking or cardiovascular disease. J. Vasc. Surg..

[26] Wallinder J, Bergqvist D, Henriksson AE (2009). Haemostatic markers in patients with abdominal aortic aneurysm and the impact of aneurysm size. Thromb. Res..

[27] Trachet B (2015). Dissecting abdominal aortic aneurysm in Ang II-infused mice: suprarenal branch ruptures and apparent luminal dilatation. Cardiovasc. Res..

[28] Kleinschnitz C (2009). Deficiency of von Willebrand factor protects mice from ischemic stroke. Blood.

[29] Zhao B-QQ (2009). von Willebrand factor-cleaving protease ADAMTS13 reduces ischemic brain injury in experimental stroke. Blood.

[30] De Meyer SF (2010). Binding of von Willebrand factor to collagen and glycoprotein Ibalpha, but not to glycoprotein IIb/IIIa, contributes to ischemic stroke in mice-brief report. Arterioscler. Thromb. Vasc. Biol..

[31] Brill A (2011). von Willebrand factor-mediated platelet adhesion is critical for deep vein thrombosis in mouse models. Blood.

[32] Gandhi C, Motto DG, Jensen M, Lentz SR, Chauhan AK (2012). ADAMTS13 deficiency exacerbates VWF-dependent acute myocardial ischemia/reperfusion injury in mice. Blood.

[33] De Meyer SF (2012). Protective anti-inflammatory effect of ADAMTS13 on myocardial ischemia/reperfusion injury in mice. Blood.

[34] Witsch, T. *et al*. Recombinant Human ADAMTS13 Treatment Improves Myocardial Remodeling and Functionality After Pressure Overload Injury in Mice. *J Am Heart Assoc* **7** (2018). 10.1161/JAHA.117.007004 PMC5850234 29367415

[35] Sénémaud J (2017). Translational Relevance and Recent Advances of Animal Models of Abdominal Aortic Aneurysm. Arterioscler. Thromb. Vasc. Biol..

[36] Deng GG (2003). Urokinase-type plasminogen activator plays a critical role in angiotensin II-induced abdominal aortic aneurysm. Circ. Res..

[37] Van Galen KP, Tuinenburg A, Smeets EM, Schutgens RE (2012). Von Willebrand factor deficiency and atherosclerosis. Blood Rev..

[38] Denis C (1998). A mouse model of severe von Willebrand disease: defects in hemostasis and thrombosis. Proc. Natl. Acad. Sci. USA.

[39] Daugherty A, Manning MW, Cassis LA (2000). Angiotensin II promotes atherosclerotic lesions and aneurysms in apolipoprotein E-deficient mice. J. Clin. Invest..

[40] Denorme F (2016). ADAMTS13-mediated thrombolysis of t-PA-resistant occlusions in ischemic stroke in mice. Blood.

[41] Daugherty A, Manning MW, Cassis LA (2001). Antagonism of AT2 receptors augments angiotensin II-induced abdominal aortic aneurysms and atherosclerosis. Br. J. Pharmacol..

[42] Portier I (2018). High and long-term von Willebrand factor expression after Sleeping Beauty transposon-mediated gene therapy in a mouse model of severe von Willebrand disease. J. Thromb. Haemost..

[43] De Cock E (2015). The novel ADAMTS13-p.D187H mutation impairs ADAMTS13 activity and secretion and contributes to thrombotic thrombocytopenic purpura in mice. J. Thromb. Haemost..

