# Supplementary material for: von Willebrand factor deficiency does not influence angiotensin II-induced abdominal aortic aneurysm formation in mice
Source: Sci Rep. 2018 Nov 9;8:16645. doi: 10.1038/s41598-018-35029-8 (PMC6226453; doi:10.1038/s41598-018-35029-8)
Supplement: Supplementary file 1 — Supplemental materials [file 41598_2018_35029_MOESM1_ESM.pdf]

von Willebrand factor deficiency does not influence angiotensin II-induced abdominal aortic aneurysm formation in mice

Irina Portier, Kimberly Martinod, Linda Desender, Nele Vandeputte, Hans Deckmyn, Karen Vanhoorelbeke, Simon F. De Meyer

SUPPLEMENTAL MATERIAL

Figure S1

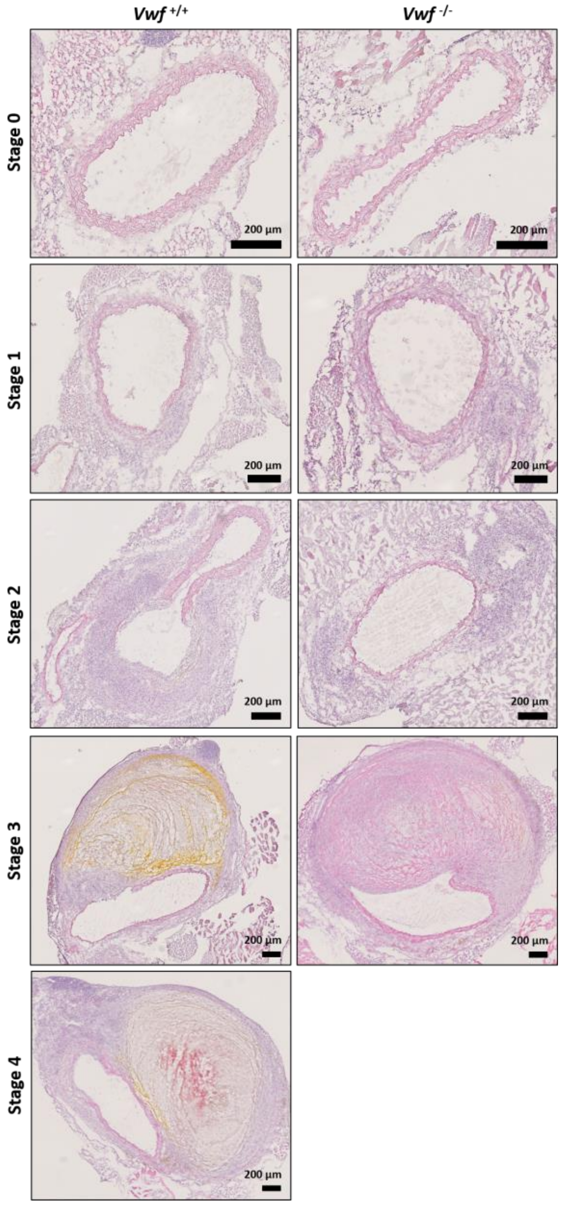

Figure S1: H&E staining of the suprarenal aortic region of *Vwf*$^{-/-}$ and *Vwf*$^{+/+}$ mice after AngII-induced AAA formation. Cryosections of suprarenal aortas of *Vwf*$^{+/+}$and *Vwf*$^{-/-}$ mice, continuously infused with AngII for 28 days, were stained with hematoxylin and eosin. Representative sections of every aneurysm stage per group are depicted. Pictures were taken using a Hamamatsu NanoZoomer-SQ digital slide scanner. AAA classification was determined as follows: Stage 0: no dilation; Stage 1: hypertrophy of the adventitia; Stage 2: dilation of the abdominal aorta with or without the presence of a thrombus; Stage 3: pronounced bulbous form of Stage 2; Stage 4: multiple, complex form of Stage 3.

Figure S2

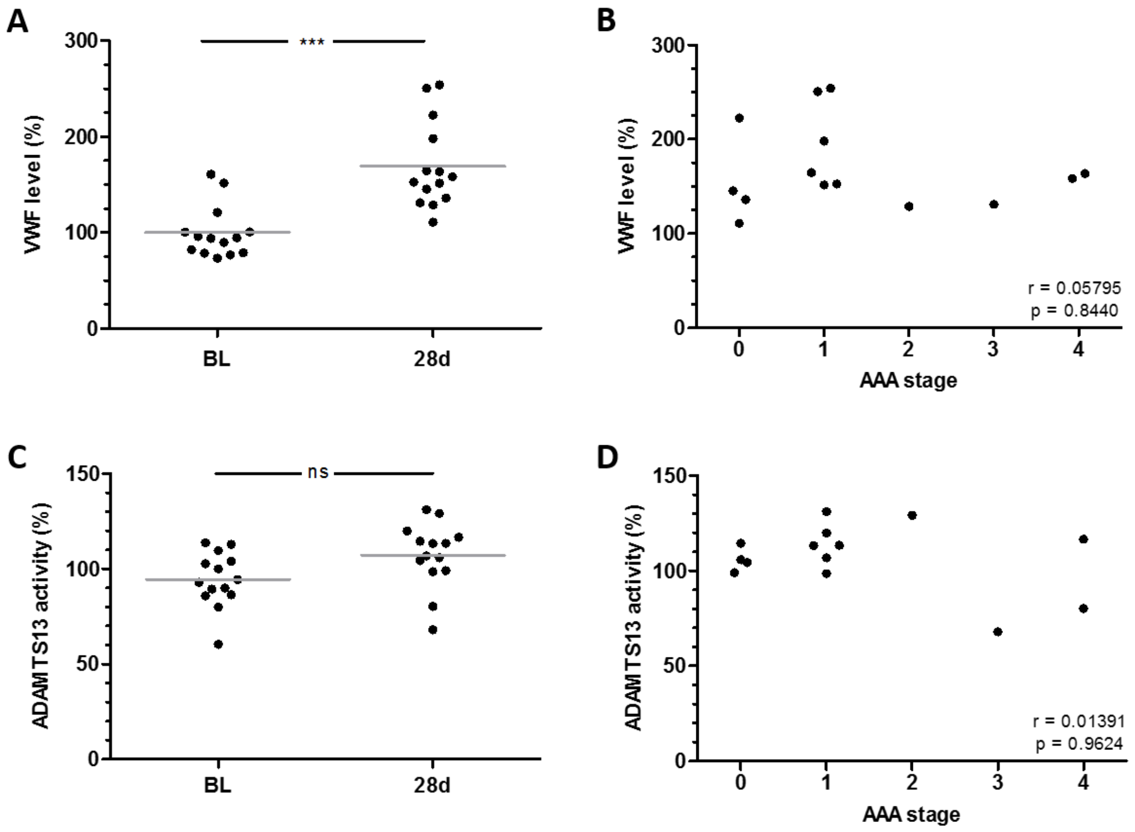

Figure S2: VWF antigen and ADAMTS13 activity in *Vwf*$^{+/+}$ mice after AngII-induced AAA formation. VWF antigen and ADAMTS13 activity levels were determined in plasma samples of *Vwf*$^{+/+}$ mice before (BL) and after 28 days (28d) of continuous AngII infusion. **(A)** VWF levels after AngII infusion were significantly increased compared to baseline levels (p=0.0001; Wilcoxon matched-pairs signed rank test). **(B)** VWF antigen levels after AngII infusion did not correlate with AAA severity (p=0.844; Spearman correlation). **(C)** ADAMTS13 activity did not change significantly after AngII infusion (p=0.053; paired t-test). **(D)** ADAMTS13 activity levels after AngII infusion did not correlate with AAA severity (p=0.962; Spearman correlation). BL, baseline: d (day), ns (not statistically significant), *** p < 0.001

Figure S3

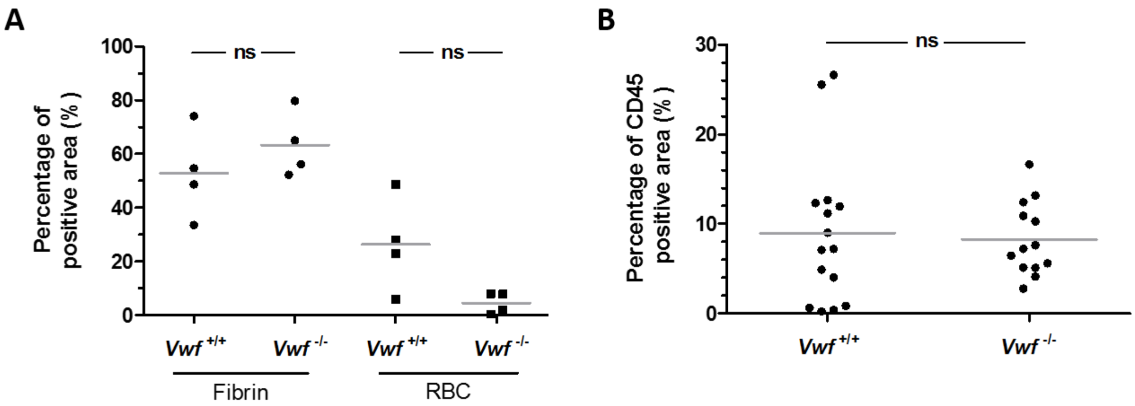

Figure S3: Quantification of the content of fibrin, red blood cells and CD45 positive cells after AngII-induced AAA formation. (A) The fibrin and red blood cell (RBC) content in the intramural thrombi of *Vwf*$^{+/+}$ or *Vwf*$^{-/-}$ mice, 28 days after continuous AngII infusion, was determined via color-based threshold analysis. Both fibrin (p=0.3429) and RBC (p=0,114; Mann-Whitney test) content were not statistically different between both groups. **(B)** Leukocyte infiltration into the suprarenal aortic tissue, was quantified by determining the percentage of CD45 positive staining by colour-based threshold analysis. No statistical difference was observed between *Vwf*$^{+/+}$ and *Vwf*$^{-/-}$ mice (p=0.782; unpaired t-test).
